# Intervention using vitamin D for elevated urinary albumin in type 2 diabetes mellitus (IDEAL-2 Study): study protocol for a randomised controlled trial

**DOI:** 10.1186/s13063-018-2616-5

**Published:** 2018-04-17

**Authors:** Shahrad Taheri, Muhammad Asim, Hassan al Malki, Omar Fituri, Manikkam Suthanthiran, Phyllis August, IDEAL-2 Study Team, Muhammad Asim, Hassan Al-Malki, Omar Fituri, Awad Rachid, Abdelaziz Adel, Ahmed Hamdi, Awais Nauman, Gamal Farghaly, Mohamad Elkadi, Shahrad Taheri, Phyllis August, Manikkam Suthanthiran, Amin Jayyousi, Buthaina Ibrahim, Ibrahim Janahi, Robert Menzies, Seleena Farook, Salma Bashir, Odette Chagoury, Sopna Choudhury, Sherryl Payra, Omar Omar, Maria Pallayova, Sahar Agouba, Sally Elgazzar, Hadya Elshakh, Hoda Gad, Samah Chalil, Amany Dahir, Hiba Tohid, Subitha Chinnaiyan

**Affiliations:** 1 Department of Medicine, Weill Cornell Medicine – Qatar, Doha, Qatar; 2 Joan and Sanford I. Weill Department of Medicine, Weill Cornell Medicine – New York, New York, USA; 3 Clinical Research Core, Weill Cornell Medicine – Qatar, Doha, Qatar; 4 Department of Medicine, Hamad Medical Corporation, Qatar Metabolic Institute (QMI), Doha, Qatar; 5 Department of Nephrology, Hamad Medical Corporation, Doha, Qatar

**Keywords:** Type 2 diabetes mellitus, Diabetic kidney disease, Albuminuria, Angiotensin converting enzyme inhibitor, Angiotensin receptor blocker, Vitamin D

## Abstract

**Background:**

The prevalence of type 2 diabetes mellitus (T2DM) is increasing worldwide. T2DM is associated with serious macro- and microvascular complications. In particular, diabetic kidney disease (DKD), which begins with excessive urinary albumin excretion, has a significant impact on affected individuals and is costly to healthcare services. Inhibition of the renin–angiotensin–aldosterone system (RAAS) with angiotensin converting enzyme inhibitors (ACEI) or angiotensin receptor blockers (ARB) significantly reduces albuminuria in diabetes, but this effect is not observed in all those treated. Active vitamin D analogues have been observed to be reno-protective through inhibition of RAAS in animal and human studies. Therefore, it can be hypothesised that an active vitamin D analogue will have an additional benefit to ACEI/ARB treatment for albuminuria reduction in DKD.

**Methods:**

The planned study is an ongoing non-blinded randomised controlled parallel-group trial examining the impact, in individuals with T2DM, of the addition of bioactive vitamin D (calcitriol) to RAAS inhibition treatment using ACI or ARB on urinary albumin excretion over a period of 26 weeks. The primary outcome measure is the urinary albumin creatinine ratio. It is planned for the study to recruit 320 participants. Other outcome measures of interest include 24-h urine albumin (24 h UA) excretion, estimated glomerular filtration rate (eGFR), blood pressure and quality of life. Safety will be assessed throughout.

**Discussion:**

If the addition of calcitriol to RAAS inhibition with ACEI or ARB safely results in a significant reduction in albuminuria, the study adds to the body of evidence supporting a role for vitamin D in reno-protection, will inform clinical practice and could result in significant reduction of healthcare costs associated with DKD.

**Trial registration:**

ISRCTN, ISRCTN86739609. Registered on 7 June 2017. ClinicalTrials.gov, NCT03216564. Registered on 13 July 2017.

**Electronic supplementary material:**

The online version of this article (10.1186/s13063-018-2616-5) contains supplementary material, which is available to authorized users.

## Background

The prevalence of type 2 diabetes mellitus (T2DM) has reached pandemic proportions. T2DM is syndemic with obesity, whose prevalence has also increased dramatically worldwide. T2DM is associated with macrovascular (ischaemic heart disease, stroke and peripheral vascular disease) and microvascular (nephropathy, retinopathy and neuropathy) complications. Of those with diabetes, 30–50% will develop kidney disease and are at risk of progressing to end-stage renal disease (ESRD) [1]. Diabetic kidney disease (DKD) is the leading cause of ESRD worldwide [1, 2]. Once ESRD occurs, there is a significant individual burden of increased cardiovascular risk and reduced quality of life as well as requirement for renal replacement therapy and transplantation with increased healthcare costs.

Renin–angiotensin–aldosterone system (RAAS) blockade with an angiotensin converting enzyme (ACE) inhibitor (ACEI) or angiotensin II type I receptor blocker (ARB) is the most widely used strategy to slow the progression of DKD in both type 1 diabetes mellitus [3] or T2DM [4, 5]. RAAS blockade reduces blood pressure, the mean rate of decline in glomerular filtration rate (GFR) by 2–10 mL/min per year and albuminuria in DKD [4, 5]. However, not all those treated achieve adequate control of blood pressure, a reduction in albuminuria or prevention of a decline of renal function [6].

Several mechanisms, including polymorphisms in the ACE gene, have been proposed to account for the variability of response to RAAS therapy [7]. An emerging hypothesis is that RAAS blockade leads to compensatory stimulation of the RAAS, contributing to therapy resistance [8]. Thus, therapeutic approaches directed at suppression of the compensatory increase in the RAAS are increasingly sought [9].

Experimental and clinical evidence suggest that vitamin D may be reno-protective in chronic kidney disease (CKD), including DKD [10–15]. In animal models of glomerular injury, 1,25-dihydroxycholecalciferol (1,25-(OH)_2_-D3; calcitriol) ameliorates glomerular injury [16] and enhances renal protection by suppressing transforming growth factor β (TGF-β)-induced α-smooth muscle actin production and type 1 collagen [17], and by increasing hepatocyte growth factor [17]. Vitamin D analogues have been reported to decrease glomerular podocyte loss and podocyte hypertrophy [18]. 1,25-(OH)_2_-D3 is a negative regulator of the RAAS. Vitamin D deficiency stimulates renin expression in normal mice and injection of 1,25-(OH)_2_-D3 reduces renin synthesis [18]. Rats with the remnant kidney model of CKD treated with the vitamin D_2_ analogue paricalcitol (19-nor-1,25-dihydroxyvitamin D_2_) showed reductions of 30–50% in kidney tissue messenger RNA (mRNA) and protein levels of angiotensinogen, renin, renin receptor and vascular endothelial cell factor, and amelioration of glomerular damage and proteinuria compared to untreated rats [19]. Diabetic mice lacking the vitamin D receptor develop more severe nephropathy than wild-type mice suggesting that vitamin D is reno-protective by suppressing the RAAS [19]. Zhang et al. demonstrated that combination therapy with the AT1 receptor blocker losartan and paricalcitol prevented albuminuria, restored glomerular filtration barrier structure and reduced glomerulosclerosis in diabetic mice, and that these changes were associated with suppression of intra-renal renin and angiotensin II, as well as transforming growth factor- β (TGF-β) [19, 20]. This group reported similar and long-term suppression of the RAAS and reduction of proteinuria using another vitamin D_2_ analogue, doxercalciferol, in experimental diabetic nephropathy [21]. In humans, observational data suggest an inverse relationship between serum 25-hydroxyvitamin D levels and both albuminuria [22] and progression of CKD [23]. Small, clinical studies show that vitamin D therapy may be useful in reducing glomerular injury in IgA nephropathy and CKD [24–26]; a secondary analysis of clinical trials of paricalcitol therapy suggested that this agent is associated with a reduction of proteinuria in individuals with various forms of CKD [27]. In a randomised clinical trial (VITAL study), de Zeeuw et al. reported that the addition of paricalcitol to RAAS inhibition safely reduces albuminuria in those with T2DM [28].

### Study rationale

The striking histologic, molecular and hormonal improvements observed with vitamin D in experimental kidney disease, coupled with promising preliminary clinical results observed in humans, provides the rationale for the IDEAL-2 (Intervention using vitamin D for elevated urinary albumin in type 2 diabetes mellitus) study. IDEAL-2 is a non-blinded randomised controlled trial (RCT) aiming to examine the impact of the addition of the active form of vitamin D (1,25-(OH)_2_-D3; calcitriol) to ACEI or ARB in T2DM individuals with albuminuria. Calcitriol was chosen as the active form of vitamin D because of its availability in Qatar.

### Hypothesis

Our primary hypothesis is that bioactive vitamin D (1,25-(OH)_2_-D3 [calcitriol]), added to inhibition of the RAAS, via ACE inhibition or ARB, has a differential impact on urine albumin excretion in T2DM compared to RAAS inhibition alone.

### Primary objective

The primary objective of IDEAL-2 is to examine the effect of calcitriol + ACEI or ARB therapy on urine albumin to creatinine ratio (ACR; measured in a standard clinical biochemistry laboratory) compared to ACEI or ARB therapy alone, after 26 weeks, in individuals with type 2 diabetes (T2DM).

### Secondary objectives

The secondary objectives of IDEAL-2 are to compare calcitriol + ACEI or ARB therapy to treatment with ACEI or ARB alone after 26 weeks for:

1. 24-h urine albumin (24 h UA) excretion;
2. Estimated glomerular filtration rate (eGFR);
3. Blood pressure;
4. Quality of life (measured using the EQ-5D questionnaire).

### Tertiary objectives

#### Diabetes complications

The study will examine the impact of the interventions on other diabetes complications. Neuropathy will be assessed using the Michigan Neuropathy Screening Instrument (MNSI) [29, 30] and physical examination. Retinopathy will be assessed through retinal photography. The Vicorder device will be used to assess measures of arterial stiffness [31].

#### Biomarkers

The study will examine circulating and urinary biomarkers associated with disease severity, disease progression and treatment response. A key objective is to develop urinary mRNA/microRNA (miRNA) biomarkers predictive of response to therapeutic interventions. Our assessment of urinary mRNA and miRNA may also provide mechanistic insights into the disease process as well as suggest mechanisms for the therapeutic outcomes. We will perform mRNA and miRNA profiling of urine collected before randomisation (baseline) and at 26 weeks of treatment and investigate whether urinary cell mRNA/miRNA profiles are associated with baseline albuminuria and eGFR in the individuals enrolled in the study and whether the urinary cell mRNA/miRNA profiles are associated with changes in albuminuria and eGFR following intervention. Our urinary mRNA profiling strategy has been successfully adapted by others to predict disease activity and progression of native kidney disease including diabetic nephropathy [32, 33]. We will utilise this assay for the measurement of urinary cell levels of mRNAs for proteins implicated in diabetic nephropathy (e.g. TGF-β1), mRNAs that may be regulated by vitamin D (e.g. renin) and podocyte-associated mRNAs (e.g. podocin). We have previously demonstrated the clinical utility of urinary cell profiles in renal allograft recipients [34–39].

Podocyte-specific loss of functional miRNAs in mice results in proteinuria, glomerular and tubular injury, renal failure and death [40–42]. miR-192 and miR-377, both upregulated by TGF-β1 in mesangial cells, are hyper-expressed in mouse models of diabetic nephropathy [41, 42]. Recently, TGF-β1 and β2 were shown to downregulate miR-200a, a miRNA with potential for preventing renal fibrogenesis [43]. Also, a loss of miR-192 has been associated with fibrogenesis in humans [44]. Wang et al. recently reported that urinary levels of miR-200a-200b and -429 are low in individuals with IgA nephropathy compared to healthy controls, and that ‘the degree of reduction correlated with disease activity and rate of progression’. Moreover, an inverse association of urinary expression of ZEB2 mRNA with miR-200b and that of vimentin mRNA with miR-200a was observed [45].

## Methods

IDEAL-2 is an ongoing non-blinded randomised controlled parallel-group trial examining the impact of the addition of bioactive vitamin D (calcitriol) to RAAS inhibition treatment using ACEI or ARB on urinary albumin excretion. The reported protocol follows the SPIRIT (Standard Protocol Items: Recommendations for Interventional Trials) recommendations (http://www.spirit-statement.org/; see Additional file 1: SPIRIT checklist). 

**Fig. 1:**
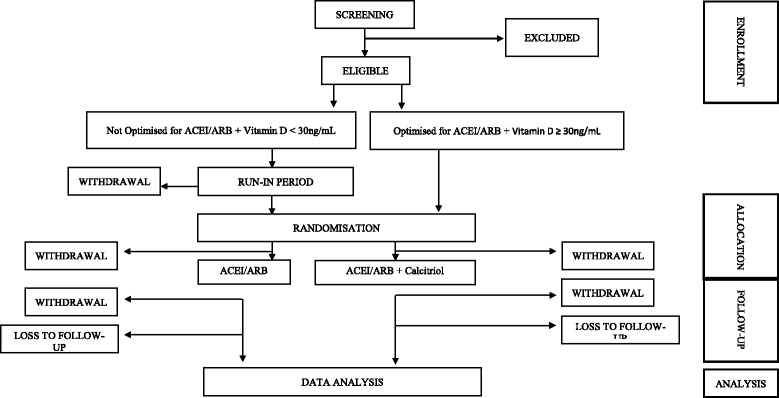
CONSORT *flow chart* for IDEAL-2 study

**Fig. 2:**
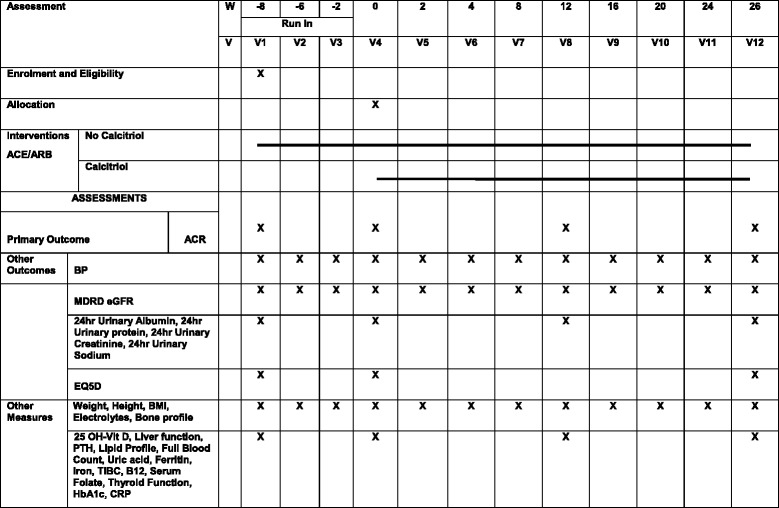
Schedule of enrolment, intervention, study visits and assessments for both study groups

Ethical approval for the study has been obtained from the Hamad Medical Corporation IRB, Weill Cornell Medicine – Qatar IRB and the Ministry of Public Health, Doha, Qatar. The study is supported by the Weill Cornell Medicine – Qatar Institutional Data and Safety Monitoring Board (DSMB) consisting of statisticians and physicians. Study audit will be conducted at three-monthly intervals by the Clinical Research Core, Weill Cornell Medicine – Qatar following a standardised protocol. The trial is registered at ISRCTN (ISRCTN86739609, date assigned 7 June 2017) and ClinicalTrials.gov (NCT03216564, registered on 13 July 2017). The description of the protocol is based on the latest version of the study protocol (PROTOCOL_IDEAL2_ST_V2.3_8JAN2017). Figure 1 shows the schedule of enrolment, intervention, study visits and assessments for the study groups. Figure 2 shows the CONSORT flow chart.

### Sponsor and funding

The study is sponsored by Hamad Medical Corporation, Qatar. The study is funded by the Qatar National Research Fund (QNRF) through the National Priorities Research Program (NPRP) grant NPRP 4–1392–3-345. Support is also provided by the Clinical Research Core at Weill Cornell Medicine in Qatar, supported by the Biomedical Research Program funded by Qatar Foundation. There is no input from the funding or sponsor organisations into the design, conduct, analysis or reporting of the study.

### Study design

IDEAL-2 is a single-centre open-label RCT, employing the following interventions (Fig. 1):

1. A[−]Calcitriol: ACEI/ARB alone (the usual standard care for diabetic albuminuria);
2. A[+]Calcitriol: ACEI/ARB + calcitriol.

### Interventions

In the A[+]Calcitriol group, participants will receive calcitriol 0.25 μg daily and optimised ACEI/ARB for 26 weeks. Adjustment of treatment will be based upon safety endpoints and if emerging data shows any evidence of safety concern.

In the A[−]Calcitriol group, participants will receive conventional therapy with ACEI/ARB for 26 weeks. Adjustment to the dosage of ACEI or ARB will be based upon safety endpoints and if emerging data show evidence of safety concern.

### Drug dosages

1. ACEI/ARB: It is expected that participants will be optimised for ACEI/ARB treatment by achieving the maximum recommended dose or, if this is not possible, the maximal tolerated dose.
2. Calcitriol: The dose of calcitriol employed in the study is 0.25 μg (250 ng) orally per day. If the calcium level is 2.62–2.79 mmol/L in the calcitriol + ACEI/ARB group and the participant is taking calcitriol 0.25 μg daily, the individual will decrease calcitriol to 0.25 μg thrice weekly. Calcium levels will be rechecked in two weeks. If the follow-up calcium level continues to be ≥ 2.62 mmol/L, the participant will discontinue use of calcitriol for the remainder of the study. If the follow-up calcium level is < 2.62 mmol/L, the individual will continue calcitriol 0.25 μg thrice weekly. If subsequently the calcium level is > 2.62 mmol/L in the calcitriol + ACEI or ARB group and the participant is taking calcitriol 0.25 μg thrice weekly, the individual will be withdrawn from the study intervention. In the unlikely event that the repeated serum calcium level is ≥ 2.62 mmol/L in the ACEI or ARB alone group at any stage, the investigator will consider withdrawing the participant from the study and investigating this clinically.

### Recruitment and setting

Individuals will be identified, screened for eligibility and recruited from the Hamad Medical Corporation outpatient clinics. Hamad Medical Corporation is the main clinical care provider in Qatar and consists of general and specialist hospitals providing secondary and tertiary care. Participants will be identified by clinicians in the outpatient department; if they are interested in participating in the study, they will be scheduled for a screening visit. The clinician will ensure that all clinically routine blood and urine tests have been conducted to allow assessment of participant eligibility and participation. Written informed consent is taken by a trained member of the research team independent of the referring physician.

### Eligibility criteria

The eligibility criteria are designed to include individuals appropriate for the study protocol. All relevant medical and non-medical conditions will be taken into consideration by the investigator team on whether the protocol is appropriate for an individual participant.

### Inclusion criteria

Individuals must meet all of the following inclusion criteria in order to be eligible for enrolment:

1. Age ≥ 18 years and < 80 years.
2. Diagnosis of T2DM requiring treatment with at least one oral hypoglycaemic medication or insulin:
  - Individuals will be considered to have established T2DM if the diagnosis of diabetes has been made and the participants were treated an oral hypoglycaemic agent or with insulin or for at least six months after diagnosis;
  - Individuals will be considered to have newly established T2DM if the diagnosis of diabetes was diagnosed with a fasting plasma glucose ≥ 7 mmol/L (126 mg/dL) or haemoglobin A1c is > 6.5% in the past six months;
  - Documented albuminuria, defined as a presence of albuminuria on two occasions in the last six months:
    i. A spot-urine ACR ≥ 30 mg/g creatinine (≥ 2.5 mg/mmol creatinine in men, ≥ 3.5 mg/mmol creatinine in women), or
    ii. Albumin ≥ 30 mg/24 h in a 24-h urine collection, or
    iii. Albumin ≥ 20 μg/min in a short-time urine collection, or
    iv. Albumin ≥ 30 mg/L in a spot-urine sample.
3. eGFR using the four-variable Modification of Diet in Renal Disease (MDRD) equation of ≥ 25 mL/min/1.73 m^2^.

### Exclusion criteria

Individuals will not be eligible for enrolment in the study if they fulfil any of the below criteria:

1. If female: positive pregnancy test or planning pregnancy in the subsequent 12 months.
2. Pregnant.
3. Breastfeeding.
4. Corrected serum calcium ≥ 2.62 mmol/L.
5. Serum potassium > 5.2 mmol/L if not on ACEI or ARB; serum potassium > 6.0 mmol/L if on ACEI or ARB.
6. 25-hydroxyvitamin D (25-OH Vit D) > 80 ng/mL.
7. PTH > 200 pg/mL.
8. Poorly controlled hypertension defined as systolic blood pressure (SBP) ≥ 180 mmHg or diastolic blood pressure ≥ 110 mmHg.
9. SBP ≤ 110 mmHg.
10. History of kidney stones.
11. History of severe chronic disease (e.g. chronic liver disease).
12. Active malignancy.
13. Recent diagnosis of acute renal failure within three months of screening visit.
14. Likelihood of renal replacement therapy within one year.
15. Any clinical and biochemical indication of primary hyperparathyroidism.
16. History of parathyroidectomy.
17. History of chronic diseases of malabsorption (e.g. Crohn’s disease, coeliac disease).
18. History of cystic fibrosis.
19. Currently taking calcitriol.
20. Currently taking calcitonin, bisphosphonates, cinacalcet, teriparatide, glucocorticoids or other drugs that may affect calcium or bone metabolism (individuals may be taking calcium containing phosphate binder or other phosphate binder. Individuals may also be taking stable dose of oestrogen/progestin).
21. Currently taking digitalis (cardiac glycosides).
22. Clinical history of osteoporosis or other bone disorder and currently on calcitriol therapy.
23. History of allergic reaction to calcitriol, paricalcitol or other 1,25-dihydroxyvitamin D analogues.
24. History of allergic reaction to any ACEI or ARB therapy.

Participation in the study will also take into account any clinical contraindications that may preclude participation as determined by the individual’s physician or the investigator team.

Those who are clinically determined to have an active infection during any urine collection will have the urine test within one week after the resolution of the infection.

### Female participants and pregnancy

To be defined as a woman of non-child-bearing potential, one of the following criteria must be met:

1. A physician documented history of hysterectomy (removal of the womb) and/or bilateral oophorectomy (removal of both ovaries); or
2. Aged > 55 years and post-menopausal (stopped menstrual periods) for > 24 months.

All other women are considered to be women of child-bearing potential. Appropriate precautions will be taken in the research study to guard against inadvertent exposure of fetuses to study drugs and to inform individuals of potential risk and the need for precautions. To minimise the possibility of fetal exposure in female participants of child-bearing potential, pregnancy testing will be performed at (screening visit) to detect unsuspected pregnancy before initiation of study treatment. If negative, the individuals must also not be planning a pregnancy for the duration of the study. All efforts will be made by the investigator to ascertain that the study participants will responsibly employ a reliable method of contraception or abstinence for the duration of the drug/treatment exposure. If requested, the investigator will refer the individual to a knowledgeable counsellor or physician for contraceptive advice. Should a participant become pregnant during the study or within three months of completing treatment, she must advise the investigator who will liaise with their obstetrician.

### Prohibited concomitant medications

Calcitonin, bisphosphonates, cinacalcet, teriparatide, glucocorticoids and other 1,25-dihydroxyvitamin D analogues, such as paricalcitol and doxercalciferol, are prohibited. If a participant has a medically necessary indication for the use of any of these medications, the individual will be required to withdraw from the study.

Participants will not be co-prescribed an ACEI and an ARB. Direct renin inhibitor use during the course of the study is prohibited. Individuals are permitted other antihypertensive agents that include but are not limited to: (1) thiazide diuretics; (2) calcium channel blockers; and (3) beta blockers. Local and international clinical guidelines for management of blood pressure in patients with DKD will be followed.

### Run-in period

The aim of the eight-week run-in period is to optimise participants for RAAS inhibition and vitamin D levels.

Participants who have T2DM and have documented albuminuria, and meet the inclusion/exclusion criteria, will be eligible for enrolment into the RCT. If not already optimised, individuals will undergo a run-in period of eight weeks during which they will be clinically optimised for ACEI or ARB treatment (the standard of care for albuminuria) and receive ergocalciferol treatment. They will then undergo randomisation to receive calcitriol + ACEI or ARB, or continue on ACEI or ARB therapy alone. Ergocalciferol treatment is discontinued at the end of the run-in period for both intervention groups.

During the 8-week run-in period, participants will discontinue current use of any direct renin inhibitor. Those who are not treated for albuminuria will be prescribed ACEI or ARB and titrated up to the recommended or highest tolerated dose. Participants’ 25-hydroxyvitamin D levels will also be measured at the start of the run-in period and, if < 30 ng/mL, they will follow the current clinical approach for vitamin D replacement which includes receiving 50,000 units of oral Vitamin D2 up to three times per week for eight weeks during the run-in period.

Individuals will be monitored for adverse effects during the run-in period. Those who tolerate ACEI or ARB and vitamin D2, if prescribed at screening, and who show adherence of > 80% with the study protocol, will then be randomised after the run-in period. Participants will be assigned to either calcitriol + ARB or ACEI group or ARB or ACEI alone group.

Throughout the run-in and study period, individuals will be treated with a goal of achieving a blood pressure target of ≤ 130/80 mmHg. Additional antihypertensive agents may be prescribed as needed. However, participants will not be prescribed a direct renin inhibitor.

Individuals will be withdrawn from the study before randomisation if the following are observed:

1. SBP ≤ 110 mmHg.
2. Serum creatinine increase > 25% of the baseline level (study entry).
3. Confirmed serum potassium > 6.0 mmol/L.
4. Corrected serum calcium ≥ 2.62 mmol/L.

Participants who are on maximal recommended ACEI or ARB therapy and have 25-hydroxyvitamin D ≥ 30 ng/mL can be directly randomised after screening without a need for the run-in period.

### Visits

Individuals will either be required to return to the study site for the outpatient visits and/or have follow-up phone calls for safety and other evaluations post randomisation at weeks 2, 4, 8, 12, 16, 20, 24 and 26.

### Participant withdrawal and safety measures

The investigators will determine whether a participant will withdraw due to an adverse event. At any point in time, the individuals can withdraw from the study. Safety measures include blood pressure and serum creatinine, potassium and corrected calcium. The key withdrawal and safety criteria include:

1. SBP ≤ 110 mmHg.
2. Serum creatinine increase > 25% of the baseline level (study entry).
3. Confirmed serum potassium > 6.0 mmol/L.
4. Corrected serum calcium ≥ 2.80 mmol/L on one test or repeated corrected serum calcium is 2.62–2.79 mmol/L, despite dose adjustment
5. Women who become pregnant during the study will be withdrawn with review to ensure safety for the mother and fetus.

### Adherence

Adherence to ACEI or ARB, vitamin D2 and calcitriol will be assessed by verbal assessment based on participant’s reported compliance. Individuals who have consistently shown significant non-compliance/non-adherence will be evaluated by the investigator for possible withdrawal from the protocol.

### Case report forms and data entry

As part of the research, participants will be asked for permission to obtain their clinical information from their medical records. Paper case report forms (CRFs) will be completed for each individual for each particular visit. Each CRF will be signed by a research investigator in order to certify that the information on the protocol is valid. The CRFs will be translated into an electronic data. All data will be double entered into a secure electronic database in preparation for data analysis. The CRF will not contain identifiable data.

### Sample size calculations

A sample size of 320 individuals will be enrolled in the study. Based on the VITAL study, we estimate a treatment effect for albuminuria of d = 0.25 sd and an intraclass correlation coefficient of 75% for our current study. To achieve a power of 80% to detect a statistically significant treatment effect for a two-tailed test with α = 0.05, we calculate a minimum of 256 participants. To cater for a potential dropout of 20%, we will enrol a total of 320 individuals.

### Randomisation and allocation concealment

Participants will be randomly allocated to receive either ACEI/ARB alone or ACEI/ARB + calcitriol. Allocation will be made in a 1:1 ratio via a web-based system that uses a computer-generated randomisation list with variable block sizes (2, 4 and 8). The allocations are computer generated in Stata (version 13.1) by the trial statistician and maintained in a secure database to which the trial coordinating team and investigators have no access. The randomisation sequence will be stratified by SBP (BP ≤ 130 mmHg and > 130 mmHg) and diabetes duration (≤ 5 years and > 5 years). Once a participant is eligible for randomisation, the research coordinator will log onto the randomisation website (https://www.sealedenvelope.com/) and perform the randomisation.

### Statistical analysis

Demographic factors and clinical characteristics will be summarised with counts (percentages) for categorical variables, mean (standard deviation [SD]) for normally distributed continuous variables or median (interquartile [IQR] or entire range) for other continuous variables.

The primary outcome is beneficial change in the primary outcome measure (ACR [log transformed]) from baseline to 26 weeks post randomisation. Primary analysis will be assessed using repeated measures analysis of covariance using a mixed model which will take account of the within-subject variability, using ACR measurements at all post-randomisation time points and adjusting for baseline ACR level and stratification factors (SBP and diabetes duration). The adjusted mean group differences for baseline and each time point with 95% confidence intervals will be calculated. Both the crude unadjusted and adjusted estimates will be presented, but the primary inference will be based on the adjusted analysis.

Secondary outcomes will be analysed using similar methods. If the outcome is skewed, then appropriate transformations will be performed. Both the primary outcome and secondary outcomes will be analysed using the intention-to-treat principle. Findings will be considered to be statistically significant at the 5% level. Statistical analyses will be performed using Stata Special Edition Version 15.0 (StataCorp LP, College Station, TX, USA).

The impact of non-response and missing data at 26 weeks post randomisation will be examined in a sensitivity analysis. In order to avoid a loss in efficiency, missing values will be imputed using multiple imputation by chained equations. Twenty imputed datasets will be created by replacing missing values with simulated values from a set of imputation models built from all potential prognostic and the outcome variable. Additional sensitivity analysis will include per protocol analysis of the results.

## Discussion

The current diabetes pandemic is likely to have serious consequences for individual health, healthcare services and society. Qatar has one of the highest prevalence of T2DM in the world which is likely to translate to a significant burden of diabetes complications. DKD is a serious complication of diabetes. Albuminuria is an early marker of DKD and is treated through improved glycaemic control, blood pressure management and RAAS inhibition. RAAS inhibition, however, does not eliminate albuminuria and progression to ESRD in all patients. There is evidence that vitamin D is a negative regulator of RAAS and may provide additional benefit to ACEI/ARB treatment.

There are several specific challenges in conducting a RCT in Qatar. The population of Qatar included expatriates who speak various languages. Because of this, the informed consent was translated into several languages (English, Arabic, Hindi, Urdu, Nepali and Malayalam) and it was imperative for the research team to speak the most common languages. Another consideration is that the expatriate community is transient, with those with greatest length of stay in Qatar being more willing to participate in clinical research [46]. This may result in participant attrition. Continuous effort will be made to ensure full participation commitment and adherence and a stringent 20% dropout was included in our sample size calculation. Challenges to successful recruitment and participation in the IDEAL-2 study will inform future clinical trials in Qatar and neighbouring countries.

The impact of active vitamin D on albuminuria has reported in a meta-analysis by de Borst et al. [47]. IDEAL-2, however, differs from recent clinical trials that have examined the impact of active vitamin D treatment on albuminuria in DKD [28, 48]. Most studies have examined the impact of paricalcitol on albuminuria. In one study, calcitriol was used in those with DKD and low eGFR at a dose of up to 0.5 μg twice weekly for 16 weeks with an observed reduction in urine protein/creatinine ratio in the calcitriol group [49]. However, no optimisation of vitamin D levels or ACEI/ARB was implemented. IDEAL-2 examines a range of albuminuria, while other studies have only included those with macro-albuminuria. IDEAL-2 also aims to optimise vitamin D levels before randomisation. This will ensure that the impact of calcitriol is independent of prior vitamin D status. IDEAL-2 includes individuals on both ACEI and ARBs who will be optimised for these drugs as well as blood pressure; other studies have used a specific ACEI or ARB. The approach in IDEAL-2 ensures greater applicability of the study findings to daily clinical practice. IDEAL-2 includes longer treatment with active vitamin D (26 weeks) than previous clinical trials, essential for assessing the durability of response. It has been proposed that active vitamin D would be a useful add-on treatment to those who are refractory to salt restriction [48]. IDEAL-2 does not stipulate any salt restriction (usually a challenge for patients) or examine in detail the impact of salt intake on urinary albumin.

If the addition of calcitriol to ACEI/ARB is observed to reduce urinary albumin, this will alter current practice. The findings from the study will be disseminated through peer-reviewed publication and results presented at international meetings to healthcare professionals. Publication authorship will follow guidelines recommended by the International Committee of Medical Journal Editors (ICMJE; http://www.icmje.org/). Further dissemination will occur through the internet and social media [50]. Ultimately, findings will be incorporated into key guidelines for treatment of patients with DKD.

## Trial status

The trial is currently underway. It is planned for the study to complete by 10 May 2019. The study follows protocol version (PROTOCOL_IDEAL2_ST_V2.3_8JAN2017). Any protocol amendments will be updated in ISRCTN and clinicaltrials.gov.

## Additional file

Additional file 1: SPIRIT 2013 Checklist. (DOC 122 kb)

## Abbreviations

24 h UA: 24-h urine albumin
ACE: Angiotensin converting enzyme
ACEI: Angiotensin converting enzyme inhibitor
ACR: Albumin-creatinine ratio
ARB: Angiotensin receptor blocker
CKD: Chronic kidney disease
CRF: Case report forms
CRP: C-reactive protein
DKD: Diabetic kidney disease
eGFR: Estimated glomerular filtration rate
EQ5D: Euroquol 5D
ESRD: End-stage renal disease
HbA1c: Glycated haemoglobin
ICMJE: International Committee of Medical Journal Editors
IgA: Immunoglobulin A
IRB: Institutional review board
MDRD: Modification of Diet in Renal Disease
miRNA: MicroRNA
MNSI: Michigan Neuropathy Screening Instrument
mRNA: Messenger RNA
NPRP: National Priorities Research Program
PTH: Parathyroid hormone
QNRF: Qatar National Research Fund
RAAS: Renin angiotensin aldosterone system
RCT: Randomised controlled trial
SBP: Systolic blood pressure
T2DM: Type 2 diabetes mellitus
TGF-β: Transforming growth factor β
TIBC: Total iron binding capacity
